# Calcium-Ion-Triggered Co-assembly of Peptide and Polysaccharide into a Hybrid Hydrogel for Drug Delivery

**DOI:** 10.1186/s11671-016-1415-8

**Published:** 2016-04-12

**Authors:** Yanyan Xie, Jun Zhao, Renliang Huang, Wei Qi, Yuefei Wang, Rongxin Su, Zhimin He

**Affiliations:** School of Environmental Science and Engineering, Tianjin University, Tianjin, 300072 People’s Republic of China; State Key Laboratory of Chemical Engineering, School of Chemical Engineering and Technology, Tianjin University, Tianjin, 300072 People’s Republic of China; Collaborative Innovation Center of Chemical Science and Engineering (Tianjin), Tianjin, 300072 People’s Republic of China; Tianjin Key Laboratory of Membrane Science and Desalination Technology, Tianjin University, Tianjin, 300072 People’s Republic of China

**Keywords:** Co-assembly, Peptide, Polysaccharide, Hybrid hydrogel, Drug delivery

## Abstract

We report a new approach to constructing a peptide–polysaccharide hybrid hydrogel via the calcium-ion-triggered co-assembly of fluorenylmethyloxycarbonyl-diphenylalanine (Fmoc-FF) peptide and alginate. Calcium ions triggered the self-assembly of Fmoc-FF peptide into nanofibers with diameter of about 30 nm. Meanwhile, alginate was rapidly crosslinked by the calcium ions, leading to the formation of stable hybrid hydrogel beads. Compared to alginate or Fmoc-FF hydrogel alone, the hybrid Fmoc-FF/alginate hydrogel had much better stability in both water and a phosphate-buffered solution (PBS), probably because of the synergistic effect of noncovalent and ionic interactions. Furthermore, docetaxel was chosen as a drug model, and it was encapsulated by hydrogel beads to study the in vitro release behavior. The sustained and controlled docetaxel release was obtained by varying the concentration ratio between Fmoc-FF peptide and alginate.

## Background

Hydrogels are a class of soft materials with attractive applications in the field of biomedicine because of their high water content, biocompatibility, and tissue-like elastic properties [1–4]. Particularly, peptide-based hydrogels fabricated by supramolecular self-assembly have received much attention because they are highly biocompatible, biodegradable in vivo, injectable, and amenable to molecular design [5–7]. To date, a wide variety of peptide-based hydrogels have been constructed from fluorenylmethyloxycarbonyl (Fmoc) peptides [8–11], peptide amphiphiles [12, 13], NAP peptides [14, 15], Fc peptides [16, 17], EAK16/RAD [18], multidomain peptides [19], and peptide polymers [20, 21]. These materials exhibit attractive prospects for application in the fields of drug delivery [22], tissue regeneration [23], three-dimensional (3D) cell cultures [24], and biosensors [7], which require certain properties such as good biocompatibility, suitable mechanical strength, high stability, environmental stimuli responsiveness, cell compatibility, and porous structures [2]. However, it is still difficult to meet all the above-mentioned demands for most of the peptide hydrogels. Therefore, the construction of a new peptide-based hydrogel with a set of desired properties is a significant topic of research.

One potential solution is to introduce other functional components into the self-assembled peptide systems to form complex hydrogels. Nanoparticles [25], graphene oxide [26], synthetic polymers [27], proteins [7, 28], and polysaccharides [22, 29] have been widely used as the functional components. For example, Stupp et al. reported the heparin-induced self-assembly of a peptide amphiphile into a polysaccharide–peptide hybrid hydrogel, which exhibited good performance in promoting angiogenesis [29]. Our group also created a self-assembling hybrid hydrogel composed of fluorenylmethyloxycarbonyl-diphenylalanine (Fmoc-FF) peptide and konjac glucomannan (KGM), Fmoc-FF–KGM, which showed much higher stability than the Fmoc-FF hydrogel, and the controlled docetaxel release from this hybrid hydrogel was also achieved [22].

The polysaccharide–protein complex widely exists in biology and contributes to many unique functions of biological organisms, such as chondroitin sulfate proteoglycan. This has greatly inspired us to develop a new peptide–polysaccharide hybrid hydrogel with a specific set of the desired properties. Herein, alginate, a kind of natural polysaccharide, is proposed as a hybrid component in view of its good hydrophilicity, high water-absorption ability, and ease of gelation through ionic crosslinking [30, 31]. However, the ionic crosslinks of this alginate hydrogel are easily broken because of ion exchange, which greatly limits its biological application. Thus, we also expect the self-assembled peptide–alginate hybrid hydrogel to have enhanced stability due to the coexistence of noncovalent (e.g., *π*–*π* stacking, hydrogen bonding) and ionic interactions when compared with peptide or alginate hydrogel alone.

In this study, we created a new peptide–polysaccharide hybrid hydrogel via calcium-ion-triggered co-assembly of Fmoc-FF peptide and alginate. The physicochemical properties including morphology, stability, and supramolecular structures were investigated. For comparison, hydrochloric acid (HCl) was introduced into a CaCl_2_ solution to trigger co-assembly of Fmoc-FF peptide and alginate, and the morphological structures of the resulting hybrid hydrogel beads were also characterized. Furthermore, the release kinetics of Fmoc-FF–alginate hydrogel beads was evaluated, using docetaxel as a drug model, for its potential application in drug delivery. We also investigated the effect of concentration ratio of Fmoc-FF and alginate on the release of docetaxel.

## Methods

### Materials

The lyophilized form of Fmoc-FF peptide was purchased from Bachem (Bubendorf, Switzerland). Alginate (*M*_w_: 32–250 kDa) was obtained from Aladdin Industrial Corp. (Shanghai, China). Docetaxel (purity >98 %) was purchased from Cenway Pharmaceutical Co. (Tianjin, China). All other chemicals, including calcium chloride, sodium hydroxide, Tween 80 (polysorbate 80), and acetonitrile, were of analytical grade and were obtained from commercial sources.

### Calcium-Ion-Triggered Self-Assembly of Fmoc-FF

In a typical experiment, 3 mg of Fmoc-FF was added to 1 mL of deionized water and homogeneously dispersed by ultrasound for 15 min, after which, it was solubilized by dropwise addition of 0.5 M NaOH. Next, 2 mL of a CaCl_2_ (2 wt.%) solution was slowly added to 1 mL of the fresh Fmoc-FF stock solution, and the mixture was aged at room temperature for 2 h without disturbance, leading to formation of transparent hydrogels.

### Calcium-Ion-Triggered Co-Assembly of Fmoc-FF and Alginate

First, fresh Fmoc-FF stock solution (3 mg/mL) was prepared by the above method. Next, 3 mg of alginate powder was slowly added to 1 mL of the fresh Fmoc-FF stock solution and stirred (1000 rpm) at room temperature for 30 min, resulting in a uniform and translucent mixed solution. The mixture was then centrifuged at 2000 rpm for 2 min to remove bubbles. Finally, the Fmoc-FF–alginate solution was added drop by drop to an abundant volume of an aqueous solution of CaCl_2_ (2.0 wt.%), using a syringe needle with an inner diameter of 0.7 mm. Hydrogel beads could be formed in a few seconds when the Fmoc-FF–alginate liquid droplets were brought into contact with the CaCl_2_ solution. In addition, we used an aqueous solution of CaCl_2_-HCl (pH = 6) as a control to study the effect of HCl on the structure of the hydrogel beads.

### Stability Test

Fmoc-FF or Fmoc-FF–alginate hydrogel beads were placed inside small vials of water or buffer solution (10-mM PBS, pH 7.4), each containing 0.1 % (*w*/*v*) Tween 80 and 0.02 % (*w*/*v*) NaN_3_. The vials were then incubated in a rotary shaker at 100 rpm and 37 °C to carry out the stability test. After 24 h, the unbroken hydrogel beads were collected by filtering and then freeze-dried. The retention of hydrogels was calculated based on following equation:

1 $$ R = W/{W}_0\times 100\% $$

where *R* represents the fraction of unbroken hydrogel at 24 h, *W* represents the mass of freeze-dried unbroken hydrogels at 24 h, *W*_0_ represents the mass of freeze-dried hydrogels before adding water or buffer solutions.

### Zeta Potential Measurement

A Zetasizer Nano ZS (Malvern Instruments Ltd., UK) was used to measure zeta potential of the Fmoc-FF peptide solutions and alginate solutions. To prepare fresh solutions, 1.5 mg of Fmoc-FF peptide was dissolved in 15 mL of double-distilled water (ddH_2_O), and 1.5 mg of alginate was dissolved in 15 mL of ddH_2_O. The pH values of the resulting solutions were adjusted with 0.5 M NaOH or 0.1 M HCl. The pH values of all the solutions were measured using a pH meter.

### In Vitro Release of Docetaxel

The release of docetaxel from different hydrogel beads was carried out in the PBS buffer at 37 °C. The docetaxel-loaded hydrogel beads were prepared according to the following protocol: Docetaxel was first dissolved in the solvent dimethyl sulfoxide (DMSO) with a concentration of 45 mg/mL. Next, 20 μL of the docetaxel-loaded DMSO solution was added to 3 mL of the fresh Fmoc-FF or Fmoc-FF–alginate stock solution, and the resulting mixture was added to 10 mL of an aqueous solution of CaCl_2_ (2.0 wt.%) using a syringe needle with an inner diameter of 0.7 mm to form drug-loaded hydrogel beads. The docetaxel-loaded hydrogel beads were then placed inside small vials containing 8 mL of PBS solution (10 mM PBS, pH 7.4), 0.1 % (*w*/*v*) Tween 80, and 0.02 % (*w*/*v*) NaN_3_. The vials were incubated in a rotary shaker at 100 rpm and 37 °C to perform the drug release experiments. An aliquot of 0.5 mL of the supernatant solution was removed from each vial at the designated sampling intervals and replaced with the same amount of fresh buffer. The docetaxel concentration in the supernatant solution was analyzed by high-performance liquid chromatography (HPLC). All the samples were centrifuged at 10 000 rpm for 5 min before they were tested by the chromatograph. The HPLC measurements were performed on an Agilent 1200 HPLC system (Agilent Technologies, USA) operating with an Aglient Eclipse XDB-C18 column. The parameters used in the HPLC analysis were as follows: the mobile phase consisted of acetonitrile water (60:40, *v*/*v*); the wavelength of UV detector was set at 229 nm; flow rate was set at of 1.0 mL min^–1^; the injection volume was 20 μL.

### Characterization

The morphologies of the hydrogels were characterized using a S-4800 field-emission scanning electron microscope (SEM; Hitachi High-Technologies Co., Japan) at an acceleration voltage of 3 kV. All samples were freeze-dried and sputter-coated with platinum using an E1045 Pt-coater (Hitachi High-technologies Co., Japan) before characterization. Fourier-transform infrared (FTIR) spectra of Fmoc-FF, alginate, and Fmoc-FF–alginate hydrogels were recorded on a Nicolet-560 FTIR spectrometer (Nicolet Co., USA). The hydrogels were freeze-dried and deposited on the surface of the KBr plate, and the FTIR spectra were recorded across the range of 400–4000 cm^–1^ with 20 scans and a resolution of 4 cm^–1^.

## Results and Discussion

**Fig. 1:**
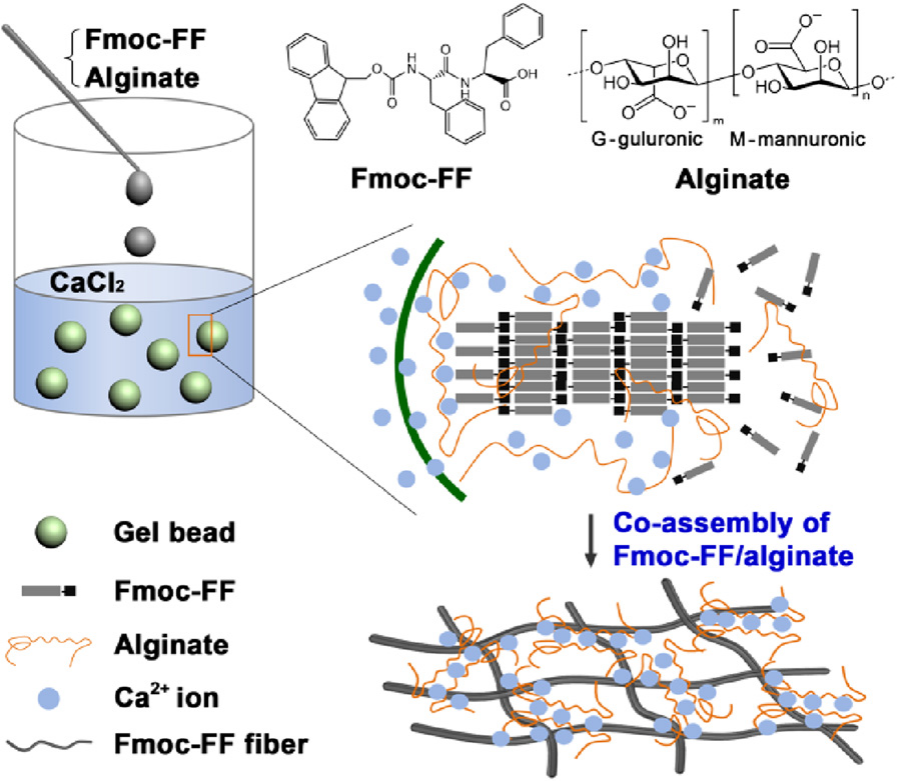
Schematic illustration of calcium-ion-triggered co-assembly of peptide and alginate at an aqueous liquid–liquid interface to synthesize Fmoc-FF–alginate hydrogel beads

In this study, we tried to fabricate a new peptide–polysaccharide hybrid hydrogel with a set of desired properties. Figure 1 illustrates the process in which calcium ions triggered co-assembly of Fmoc-FF peptide and alginate at an aqueous liquid–liquid interface to synthesize Fmoc-FF–alginate hydrogel beads. As the mixture solution was added dropwise to an aqueous solution of CaCl_2_, the alginate could be rapidly crosslinked by calcium ions. On the other hand, the Fmoc-FF molecules could self-assemble into nanofibers via noncovalent interactions because their charge was decreased by the calcium ions. The crosslinked alginate molecules were interwoven with the Fmoc-FF fibrous network, resulting in the formation of hybrid hydrogel beads.

**Fig. 2:**
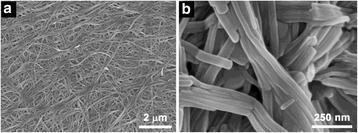
**a**-**b** SEM images of Fmoc-FF nanofibers formed in the presence of Ca^2+^ ions

We first studied the self-assembly behavior of Fmoc-FF peptide triggered by Ca^2+^ ions. As shown in Fig. 2, long nanofibers with no branching or cross-linking were observed as the framework of the Fmoc-FF peptide hydrogel, suggesting that the hydrogel morphology relied on the noncovalent interactions between the nanofibers. These nanofibers had a smaller diameter of 100 nm and higher degree of entanglement than those of nanofibers formed by HCl-triggered self-assembly of Fmoc-FF [32].

**Fig. 3:**
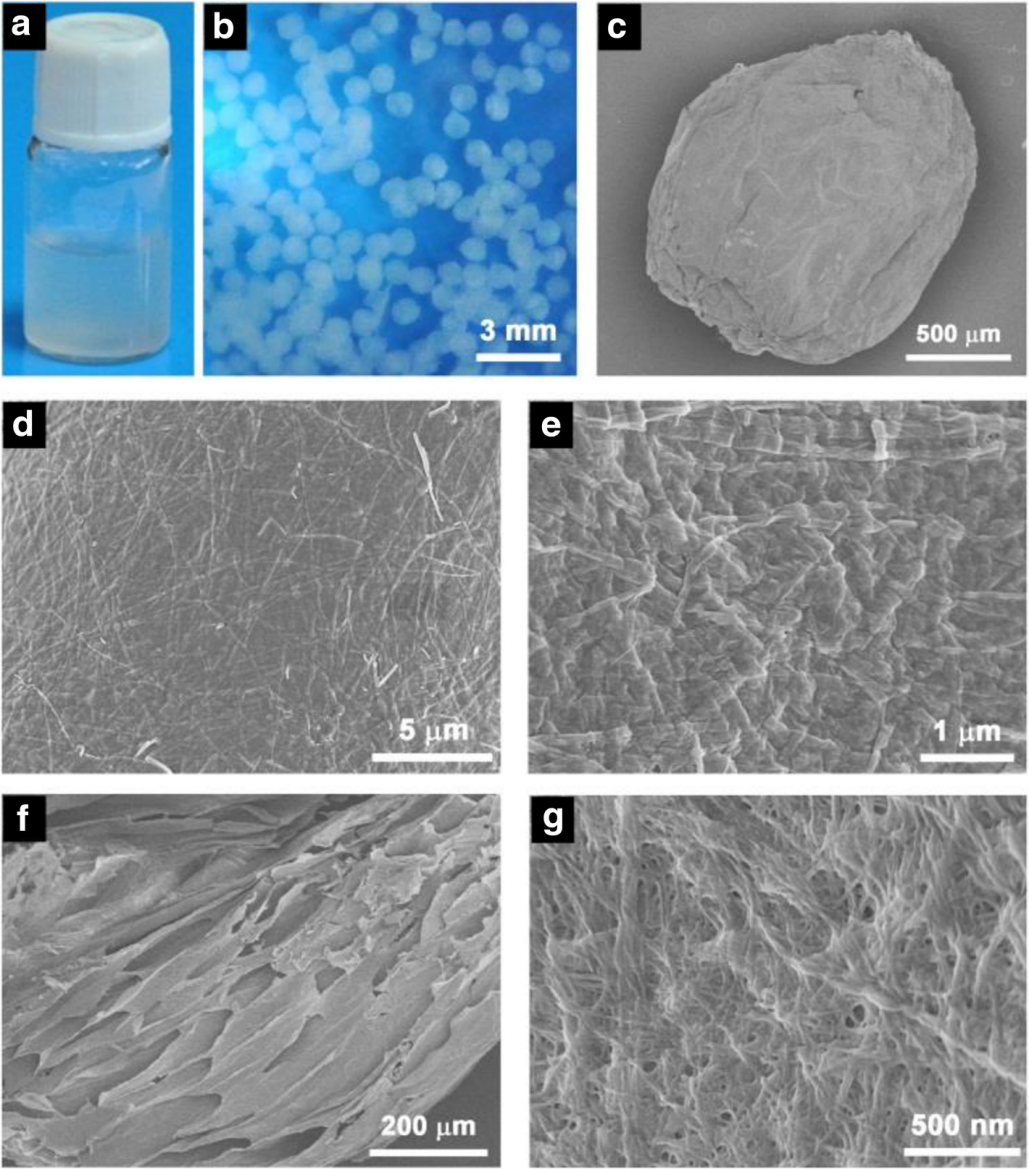
Photos of the (**a**) Fmoc-FF–alginate solution and (**b**) Fmoc-FF–alginate hydrogel beads. (**c**– **g**) SEM images of Fmoc-FF–alginate hydrogel beads formed in the presence of Ca^2+^ ions

After the initial SEM characterization, we further studied the calcium-ion-triggered co-assembly behavior of Fmoc-FF peptide and alginate. The Fmoc-FF–alginate solution (Fig. 3a). was added dropwise to an abundant volume of an aqueous solution of CaCl_2_ to trigger co-assembly. The formation of hydrogel beads was observed within a few seconds when the Fmoc-FF–alginate liquid droplets made contact with the CaCl_2_ solution. These hydrogel beads had a diameter of approximately 1 mm and showed good uniformity (Fig. 3b). To gain insight into the exterior and interior micro-morphology of the hydrogel beads, SEM was employed to observe the freeze-dried samples. As shown in Fig. 3c–g, a typical hydrogel bead had a dense surface structure on the exterior (Fig. 3c–e), while its interior showed a structure of stacked sheets (Fig. 3f) that formed a porous fibrous network (Fig. 3g). The sheets were possibly generated by freeze-drying [33]. When the Fmoc-FF–alginate liquid droplets first made contact with the CaCl_2_ solution, the alginates were rapidly crosslinked by Ca^2+^ ions, while fewer Fmoc-FF nanofibers were formed because of the low self-assembly rate of Fmoc-FF peptide, resulting in a dense gel layer at the liquid–liquid interface of the Fmoc-FF–alginate droplet and the aqueous CaCl_2_ solution. As time went on, the Ca^2+^ ions diffused into the interior of the liquid droplet and triggered co-assembly of Fmoc-FF and alginate, forming a porous nanofibrous gel network. In the co-assembly process, the Ca^2+^ ions triggered self-assembly of Fmoc-FF into nanofibers to provide a matrix for the gel beads. Meanwhile, the alginate was crosslinked by Ca^2+^ ions and then interwoven with the Fmoc-FF nanofibers, forming the hybrid hydrogel beads. The hybrid hydrogel beads consisting of Fmoc-FF nanofibers had a smaller diameter of about 30 nm and were curlier than the beads of the Fmoc-FF hydrogel without alginate. The higher viscosity of the Fmoc-FF–alginate solution may have prevented the further growth of the Fmoc-FF assembly structure, thus resulting in the formation of fine fibers.

**Fig. 4:**
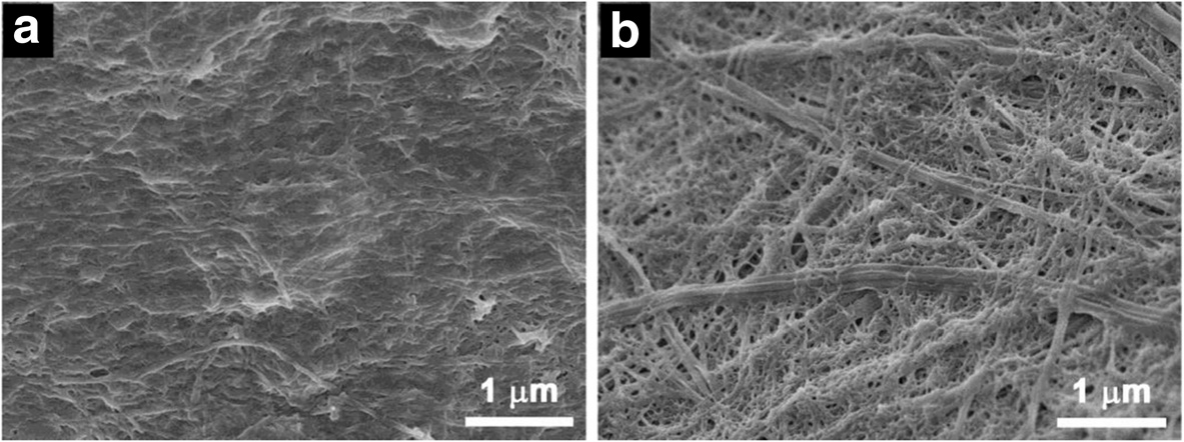
SEM images of Fmoc-FF–alginate hydrogel beads formed in (**a**) a CaCl_2_ solution and (**b**) a CaCl_2_–HCl mixed solution (pH = 6)

The microstructures of supramolecular hydrogels are controlled by the nucleation processes and growth rates. It has been reported that hydrochloric acid can induce self-assembly of Fmoc-FF into nanofibers and form a hydrogel by decreasing the charge of the molecules. Therefore, we introduced HCl into the aqueous solution of CaCl_2_ to study its effect on the structure of the hybrid hydrogel beads. As can be seen in Fig. 4a, the hydrogel beads, which were formed in the CaCl_2_ solution, had a denser surface composed of uniform nanofibers and a large amount of crosslinked alginate. For the hybrid hydrogel beads formed in the CaCl_2_–HCl solution (pH = 6), a porous fibrous network, in which Fmoc-FF nanofibers with different diameters were interwoven with the lightly crosslinked alginate, was observed on its surface (Fig. 4b), demonstrating that HCl had a significant effect on the structure of the hybrid hydrogel beads. In the absence of HCl, the alginate was quickly crosslinked by Ca^2+^ ions and fewer Fmoc-FF nanofibers were formed at liquid–liquid interface, thus resulting in a dense gel layer because of the slow self-assembly rate of Fmoc-FF peptide. In the presence of HCl, H^+^ could trigger fast self-assembly of Fmoc-FF into nanofibers with large diameters and a smaller amount of alginate was crosslinked by Ca^2+^ ions towing to the slower rate, leading to a porous fibrous network on the surface of hybrid hydrogel bead.

**Fig. 5:**
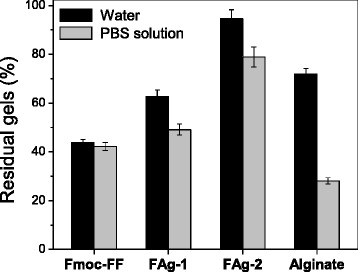
Retention of hydrogel beads in water and PBS after 24 h. FAg-1 is the Fmoc-FF–alginate hybrid hydrogel (concentration ratio of 2:1); FAg-2 is the Fmoc-FF–alginate hybrid hydrogel (concentration ratio of 1:1)

The stability of hydrogel beads was evaluated by immersing them in water and a phosphate buffer solution (PBS) with pH of 7.4, both at a temperature of 37 °C. As shown in Fig. 5, 72.2 % of the alginate hydrogel beads remained after 24 h, indicating their relatively high stability in water. However, obvious destruction (71.8 %) occurred for the alginate hydrogel beads in PBS during the same interval as a result of the ion exchange between Ca^2+^ ions and Na^+^ ions. For the Fmoc-FF hydrogel beads, 56.0 and 57.6 % of the hydrogel beads were degraded in water and PBS, respectively, which is attributed to the weakly noncovalent interactions between Fmoc-FF molecules. It is generally known that salt has little effect on the stability of Fmoc-FF hydrogel beads. The Fmoc-FF–alginate hybrid hydrogel beads exhibited high stability in both water and PBS. When the concentration ratio of Fmoc-FF to alginate was 2:1, 37.3 and 50.8 % of the hydrogel beads were broken up in water and PBS, respectively. When the Fmoc-FF/alginate concentration ratio was reduced to 1:1, only a small amount of breakage (5.5 % in water and 21.1 % in PBS) occurred among the hybrid hydrogel beads. For the Fmoc-FF hydrogel beads, the bead retention increased from 44.0 to 94.5 % as the alginate amount was varied from 0 to 3 mg/mL in water, demonstrating that the introduction of alginate could enhance the stability of the Fmoc-FF hydrogel. For the alginate hydrogel, the retention increased from 28.2 to 78.9 % as the Fmoc-FF concentration was varied from 0 to 3 mg/mL in PBS. In addition, self-assembled Fmoc-FF nanofibers improved the salt tolerance of the alginate hydrogel. Combining the findings of enhanced stability in water and salt tolerance in the PBS buffer, we could infer that the synergistic effect of noncovalent (e.g., π–π stacking, hydrogen bonding) and ionic interactions contributed to the high stability of the Fmoc-FF–alginate hybrid hydrogels.

**Fig. 6:**
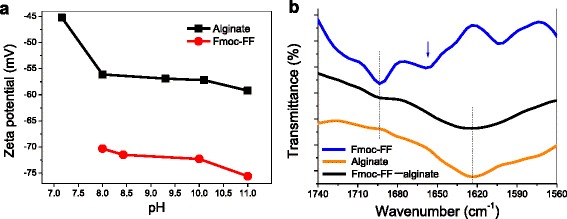
(**a**) Zeta potential of Fmoc-FF and alginate molecule in water. (**b**) FTIR spectra of Fmoc-FF hydrogel, alginate hydrogel, and Fmoc-FF–alginate hybrid hydrogel

The zeta potentials of Fmoc-FF peptide and alginate were measured at different pH values to analyze the deprotonated degree of carboxylic acid and hydroxyl (Fig. 6a). Because Fmoc-FF molecule can self-assemble into nanofibers at pH 7–8 and it is easily hydrolyzed at pH >11, the zeta value was only measured over the pH range of 8 to 11 for Fmoc-FF peptide. In an alkaline environment, the Fmoc-FF peptide and alginate molecules are expected to be ionized and they all displayed negative zeta potentials below –30 mV. Interestingly, a jump of the zeta value was observed for the alginate as the pH value was increased from 7 to 8, which is related to the deprotonation of hydroxyl groups. Fmoc-FF peptide and alginate showed zeta potentials of –57 and –73 mV, respectively, in their mixed solutions, indicating that they were stable in the solutions. It further demonstrates that the high stability of the hybrid hydrogel beads is attributed to calcium ion crosslinking and intertwining of the Fmoc-FF nanofibers with alginate. The molecular arrangement within the hydrogel beads was characterized using FTIR. Figure 6b shows the FTIR spectra of Fmoc-FF hydrogel, alginate hydrogel, and Fmoc-FF–alginate hybrid hydrogel. Two characteristic absorption peaks appear at 1657 and 1693 cm^−1^ in the amide I region for the Fmoc-FF hydrogel, suggesting an anti-parallel β-sheet arrangement of Fmoc-FF peptide [22, 32]. Alginate shows one strong and broad absorption peak at 1625 cm^−1^, which is attributed to the large amount of the carboxylic acid group (–COO^−^). For the hybrid hydrogel, one obvious peak appears at 1692 cm^−1^ and a relatively broad peak appears at 1625 cm^−1^, indicating that alginate did not change the molecular arrangement of Fmoc-FF peptide in the co-assembly process.

**Fig. 7:**
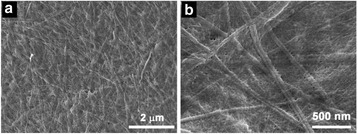
(**a**-**b**) SEM images of the docetaxel-loaded Fmoc-FF–alginate hydrogel beads

The high stability of these hydrogel beads may allow them to deliver good performance in drug delivery. Docetaxel was chosen as a drug model, and docetaxel-loaded hydrogel beads were prepared to investigate how the drug would be released. As shown in Fig. 7, no obvious change was seen in the morphology of the hybrid hydrogel beads after the docetaxel was encapsulated.

**Fig. 8:**
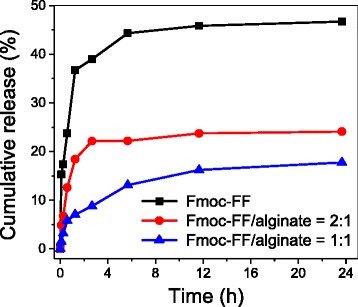
Cumulative release of docetaxel from drug-loaded Fmoc-FF hydrogel beads and drug-loaded Fmoc-FF–alginate hydrogel beads with different alginate concentrations

The in vitro release behavior of the docetaxel from the Fmoc-FF–alginate hydrogel beads was then studied using Fmoc-FF hydrogel beads as a control. As shown in Fig. 8, the release of docetaxel from the Fmoc-FF hydrogel beads was remarkably faster than that from the Fmoc-FF–alginate hydrogel beads. A burst release occurred among the Fmoc-FF hydrogel beads, where ~46 % of docetaxel was released within 24 h, while only 24 % of docetaxel was released from the Fmoc-FF–alginate hydrogel beads (concentration ratio of Fmoc-FF/alginate ratio = 2:1) over the same period. In addition, the cumulative release of docetaxel from the Fmoc-FF–alginate hydrogel beads was reduced to 15 % as the alginate concentration reached 3 mg/mL within 24 h, indicating that the existence of alginate could restrain the quick release of docetaxel from the hydrogel beads. The slower drug release rate from the Fmoc-FF–alginate hydrogel beads may have been related to their more stable hydrogel structure. By changing the concentration ratio of Fmoc-FF to alginate, sustained and controlled release of docetaxel could be obtained.

In order to better understand the release mechanism of docetaxel from the hydrogel beads, the following Korsmeyer–Peppas equation was employed to fit the release profiles: [34, 35]

2 $$ {M}_{\mathrm{t}}/{M}_{\infty }=k{t}^n $$

3 $$ lg\;\left(\mathrm{release}\%\right)= lg\;\left({M}_{\mathrm{t}}/{M}_{\infty}\right)=n\; lg\;t+ lg\;k $$

where *M*_t_/*M*_∞_ represents the fraction of drug released from hydrogel beads at time *t*, *k* represents the kinetic constant characteristic of the drug/hydrogel matrix, and *n* is the diffusional exponent characteristic of the release mechanism. When *n* = 0.5, the drug release is purely controlled by Fickian diffusion; when 0.5 < *n* < 1, more than one mechanism is at work; when *n* = 1, the drug release is controlled by hydrogel swelling (case II transport).

**Fig. 9:**
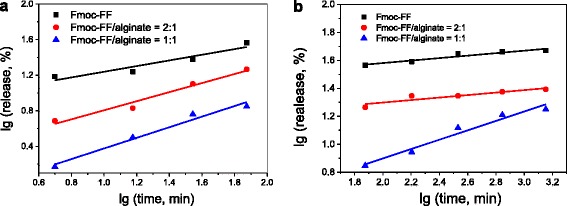
Plots of lg (release, %) against lg (time, min) for docetaxel release from drug-loaded Fmoc-FF–alginate hydrogel beads: (**a**) 0-1.25 h; (**b**) 1.25 -24 h.

Figure 9 shows the linear fit of our experimental data for docetaxel release from hydrogel beads based on Eq. 3. A summary of the values of the release exponent (*n*), rate constant (*k*), and squared correlation coefficient (*R*^2^) is presented in Table 1. For the Fmoc-FF–alginate hybrid hydrogel beads, we obtained a good linear fit of the experimental data of docetaxel release. For the Fmoc-FF hydrogel beads, the fitting result was poor because of their structure instability. In the beginning stage of drug release (0–1.25 h), the exponent *n* = 0.3184 for Fmoc-FF hydrogel beads indicates that docetaxel was mainly released in a Fickian diffusion-mediated mode. The value of *n* < 0.5 might be attributable to the hydrophobic interaction between Fmoc-FF nanofibers and docetaxel. For the hybrid hydrogel beads, the *n* values are close to 0.5, indicating a Fickian diffusion-controlled process for docetaxel release. The value of exponent *n* increases with the increasing alginate concentration, which might be due to the fact that the hydrophilic alginate could weaken the hydrophobic interaction between Fmoc-FF nanofibers and docetaxel, and thus lead to faster drug release. In the late stage of drug release (1.25–24 h), *n* = 0.0885 for Fmoc-FF hydrogel beads, and *n* = 0.0890 for the hybrid hydrogel beads (Fmoc-FF/alginate = 2:1). There are two reasons for these values: the encapsulation of docetaxel in a deeper part of the hydrogel beads; hydrophobic interaction between Fmoc-FF nanofibers and docetaxel may have been responsible for the slow release behavior. Thus, the Korsmeyer–Peppas empirical model is no longer suitable for docetaxel release from these hydrogel beads in this stage. However, the value of *n* was 0.3359 for hybrid hydrogel beads with an alginate concentration of 3 mg/mL, indicating that the docetaxel release continued to exhibit Fickian diffusion-mediated behavior. It also indicates that the release of docetaxel from the hydrogel beads took longer when the amount of alginate in these hydrogel beads was increased. Moreover, the highly stable structure of hydrogel caused by co-assembly resulted in release behavior that was close to the empirical model.

**Table 1:** Release exponent (*n*), rate constant (*k*), and correlation coefficient (*R* ^2^) obtained from the power law equation

Hydrogels	0–1.25 h			1.25–24 h		
	*n*	*k*	*R* ^2^	*n*	*k*	*R* ^2^
Fmoc-FF	0.3184	8.3163	0.8538	0.0885	25.326	0.9037
Fmoc-FF/alginate = 2	0.5094	1.9773	0.9565	0.0890	13.191	0.8036
Fmoc-FF/alginate = 1	0.5962	0.6024	0.9605	0.3359	1.6846	0.9518

## Conclusions

A new peptide–polysaccharide hybrid hydrogel bead was prepared by calcium ion-triggered co-assembly of Fmoc-FF peptide and alginate. The calcium ions triggered self-assembly of Fmoc-FF peptide into nanofibers with a diameter of about 30 nm by decreasing their charge; at the same time, they allowed alginate to be crosslinked, leading to the formation of hybrid hydrogel beads. SEM results verified that the hybrid hydrogel beads had a dense surface structure on the outside and a structure of stacked sheets with a porous nanofibrous network in its interior. Compared to the alginate or Fmoc-FF hydrogel beads, the hybrid hydrogel beads had higher stability in both water and PBS, probably because of the synergistic effect of noncovalent and ionic interactions. Furthermore, we embedded docetaxel in the hydrogel beads during the hydrogel formation process and studied the drug release behavior. By varying the concentration ratio of Fmoc-FF to alginate, controlled release of docetaxel could be obtained. The release exponent *n* suggests that docetaxel was mainly released from the hybrid beads in a Fickian diffusion-mediated mode. We thus conclude that our method provides a way to fabricate new supramolecular materials with fascinating properties.

## References

[1] Wang Y, Li B, Zhou Y, Jia D (2009). In situ mineralization of magnetite nanoparticles in chitosan hydrogel. Nanoscale Res Lett.

[2] Seliktar D (2012). Designing cell-compatible hydrogels for biomedical applications. Science.

[3] Appel EA, Loh XJ, Jones ST, Biedermann F, Dreiss CA, Scherman OA (2012). Ultrahigh-water-content supramolecular hydrogels exhibiting multistimuli responsiveness. J Am Chem Soc.

[4] Geng H, Song H, Qi J, Cui D (2011) Sustained release of VEGF from PLGA nanoparticles embedded thermo-sensitive hydrogel in full-thickness porcine bladder acellular matrix. Nanoscale Res Lett. 6:312-319 10.1186/1556-276X-6-312 PMC3211399 21711840

[5] Scott CM, Forster CL, Kokkoli E (2015). Three-dimensional cell entrapment as a function of the weight percent of peptide-amphiphile hydrogels. Langmuir.

[6] Li J, Gao Y, Kuang Y, Shi J, Du X, Zhou J, Wang H, Yang Z, Xu B (2013). Dephosphorylation of D-peptide derivatives to form biofunctional, supramolecular nanofibers/hydrogels and their potential applications for intracellular imaging and intratumoral chemotherapy. J Am Chem Soc.

[7] Ikeda M, Tanida T, Yoshii T, Kurotani K, Onogi S, Urayama K, Hamachi I (2014). Installing logic-gate responses to a variety of biological substances in supramolecular hydrogel-enzyme hybrids. Nat Chem.

[8] Nguyen MM, Eckes KM, Suggs LJ (2014). Charge and sequence effects on the self-assembly and subsequent hydrogelation of Fmoc-depsipeptides. Soft Matter.

[9] Orbach R, Adler-Abramovich L, Zigerson S, Mironi-Harpaz I, Seliktar D, Gazit E (2009). Self-assembled Fmoc-peptides as a platform for the formation of nanostructures and hydrogels. Biomacromolecules.

[10] Xie Y, Huang R, Qi W, Wang Y, Su R, He Z (2016). Enzyme-substrate interactions promote the self-assembly of amino acid derivatives into supramolecular hydrogels. J Mater Chem B.

[11] Xie Y, Wang X, Huang R, Qi W, Wang Y, Su R, He Z (2015). Electrostatic and aromatic interaction-directed supramolecular self-assembly of a designed Fmoc-tripeptide into helical nanoribbons. Langmuir.

[12] Tanaka A, Fukuoka Y, Morimoto Y, Honjo T, Koda D, Goto M, Maruyama T (2015). Cancer cell death induced by the intracellular self-assembly of an enzyme-responsive supramolecular gelator. J Am Chem Soc.

[13] Webber MJ, Tongers J, Renault M-A, Roncalli JG, Losordo DW, Stupp SI (2010). Development of bioactive peptide amphiphiles for therapeutic cell delivery. Acta Biomater.

[14] Tian Y, Wang H, Liu Y, Mao L, Chen W, Zhu Z, Liu W, Zheng W, Zhao Y, Kong D, Yang Z, Zhang W, Shao Y, Jiang X (2014). A peptide-based nanofibrous hydrogel as a promising DNA nanovector for optimizing the efficacy of HIV vaccine. Nano Lett.

[15] Liu J, Liu J, Chu L, Zhang Y, Xu H, Kong D, Yang Z, Yang C, Ding D (2014). Self-assembling peptide of D-amino acids boosts selectivity and antitumor efficacy of 10-hydroxycamptothecin. ACS Appl Mater Interfaces.

[16] Sun Z, Li Z, He Y, Shen R, Deng L, Yang M, Liang Y, Zhang Y (2013). Ferrocenoyl phenylalanine: a new strategy toward supramolecular hydrogels with multistimuli responsive properties. J Am Chem Soc.

[17] Wang Y, Qi W, Huang R, Yang X, Wang M, Su R, He Z (2015). Rational design of chiral nanostructures from self-assembly of a ferrocene-modified dipeptide. J Am Chem Soc.

[18] Yang Y, Khoe U, Wang X, Horii A, Yokoi H, Zhang S (2009). Designer self-assembling peptide nanomaterials. Nano Today.

[19] Jiang L, Xu D, Sellati TJ, Dong H (2015). Self-assembly of cationic multidomain peptide hydrogels: supramolecular nanostructure and rheological properties dictate antimicrobial activity. Nanoscale.

[20] Perez CMR, Rank LA, Chmielewski J (2014). Tuning the thermosensitive properties of hybrid collagen peptide-polymer hydrogels. Chem Commun.

[21] Ponnumallayan P, Fee CJ (2014). Reversible and rapid ph-regulated self-assembly of a poly(ethylene glycol)-peptide bioconjugate. Langmuir.

[22] Huang R, Qi W, Feng L, Su R, He Z (2011). Self-assembling peptide-polysaccharide hybrid hydrogel as a potential carrier for drug delivery. Soft Matter.

[23] Cheng T-Y, Chen M-H, Chang W-H, Huang M-Y, Wang T-W (2013). Neural stem cells encapsulated in a functionalized self-assembling peptide hydrogel for brain tissue engineering. Biomaterials.

[24] Szkolar L, Guilbaud J-B, Miller AF, Gough JE, Saiani A (2014). Enzymatically triggered peptide hydrogels for 3D cell encapsulation and culture. J Pept Sci.

[25] Altunbas A, Sharma N, Lamm MS, Yan C, Nagarkar RP, Schneider JP, Pochan DJ (2010). Peptide-silica hybrid networks: biomimetic control of network mechanical behavior. ACS Nano.

[26] Wu J, Chen A, Qin M, Huang R, Zhang G, Xue B, Wei J, Li Y, Cao Y, Wang W (2015). Hierarchical construction of a mechanically stable peptide-graphene oxide hybrid hydrogel for drug delivery and pulsatile triggered release in vivo. Nanoscale.

[27] Maslovskis A, Guilbaud JB, Grillo I, Hodson N, Miller AF, Saiani A (2014). Self-assembling peptide/thermoresponsive polymer composite hydrogels: effect of peptide-polymer interactions on hydrogel properties. Langmuir.

[28] Zhang X, Dong C, Huang W, Wang H, Wang L, Ding D, Zhou H, Long J, Wang T, Yang Z (2015). Rational design of a photo-responsive UVR8-derived protein and a self-assembling peptide-protein conjugate for responsive hydrogel formation. Nanoscale.

[29] Abul-Haija YM, Ulijn RV (2015). Sequence adaptive peptide-polysaccharide nanostructures by biocatalytic self-assembly. Biomacromolecules.

[30] Kuo CK, Ma PX (2001). Ionically crosslinked alginate hydrogels as scaffolds for tissue engineering: Part 1. Structure, gelation rate and mechanical properties. Biomaterials.

[31] McConaughy SD, Kirkland SE, Treat NJ, Stroud PA, McCormick CL (2008). Tailoring the network properties of Ca2+ crosslinked aloe vera polysaccharide hydrogels for in situ release of therapeutic agents. Biomacromolecules.

[32] Smith AM, Williams RJ, Tang C, Coppo P, Collins RF, Turner ML, Saiani A, Ulijn RV (2008). Fmoc-diphenylalanine self assembles to a hydrogel via a novel architecture based on π-π interlocked β-sheets. Adv Mater.

[33] Deville S, Saiz E, Nalla RK, Tomsia AP (2006). Freezing as a path to build complex composites. Science.

[34] Ritger PL, Peppas NA (1987). A simple equation for description of solute release II. Fickian and anomalous release from swellable devices. J Control Release.

[35] Hassan MM, Martin AD, Thordarson P (2015). Macromolecular crowding and hydrophobic effects on Fmoc-diphenylalanine hydrogel formation in PEG : water mixtures. J Mater Chem B.

